# *Lactobacillus paragasseri* SBT2055 Activates Plasmacytoid Dendritic Cells and Improves Subjective Symptoms of Common Cold in Healthy Adults: A Randomized, Double-Blind, Placebo-Controlled Parallel-Group Comparative Trial

**DOI:** 10.3390/nu15204458

**Published:** 2023-10-20

**Authors:** Eiji Kobatake, Yoshitaka Iwama, Toshinobu Arai, Yuki Tsukisaka, Toshihide Kabuki

**Affiliations:** 1 Milk Science Research Institute, MEGMILK SNOW BRAND Co., Ltd., Kawagoe 350-1165, Japan; 2 Nihonbashi Cardiology Clinic, Tokyo 103-0001, Japan; 3 Research and Development Planning Department, MEGMILK SNOW BRAND Co., Ltd., Tokyo 160-8575, Japan; 4 KSO Corporation, Tokyo 105-0023, Japan

**Keywords:** *Lactobacillus paragasseri* SBT2055, clinical study, subjective symptoms, common cold, plasmacytoid dendritic cells

## Abstract

This study investigated whether *Lactobacillus paragasseri* SBT2055 (LG2055) activates plasmacytoid dendritic cells (pDCs) and suppresses common cold symptoms in healthy adults. Cell-based experiments showed that a LG2055 treatment upregulated CD86 and HLA-DR expression in pDCs, indicating that LG2055 activates pDCs in vitro. In a subsequent randomized, double-blind, placebo-controlled, parallel-group comparative trial, 200 participants were randomly divided into two groups and consumed three capsules with or without LG2055 once daily for 12 weeks. The primary outcome was the score on a daily physical health questionnaire survey of common cold symptoms. Three participants discontinued the trial and six participants were excluded from the analysis, thus 191 participants (95 in the LG2055 group and 96 in the placebo group) were analyzed. The LG2055 group showed a significantly higher ratio of “without symptoms” responses for runny nose, plugged nose, sneezing, sore throat, hoarseness, and chill than the placebo group. Furthermore, a stratified analysis revealed that LG2055 intake enhanced CD86 and HLA-DR expression in the pDCs of the participants with low secretion rates of salivary secretory immunoglobulin A. These data suggest that LG2055 suppresses the subjective symptoms of the common cold by activating pDCs and improving the host’s immune system in healthy adults, especially in immune-weakened individuals (UMIN000049183).

## 1. Introduction

The coronavirus disease 2019 (COVID-19) pandemic has greatly changed the public’s mindset toward infectious diseases. Humans have always been threatened by the spread of new viral infections, such as the 2009 influenza virus A (H1N1) pandemic [1], while the risk of infection by known viruses or pathogens represents another threat. Moreover, several factors such as stress, poor sleep, or exhaustion weaken the immune system and increase the risk of infection [2,3,4]. Maintenance and/or an enhancement of the host’s biological defense system is essential to avoid such infections.

Numerous cell types and humoral factors contribute to the immune system. Plasmacytoid dendritic cells (pDCs), a rare immune cell type, have received extensive research attention in this regard recently. pDCs are important for the response to viral infections. Activated pDCs produce large amounts of type I interferons and activate many types of immune cells, such as natural killer (NK) cells, B cells, and T cells, which enhance the immune system and protect the host from infection [5]. Although pDC activation is usually induced by the recognition of viral DNA or RNA during viral infection [5], agents that activate pDCs can be used to enhance the host’s defense system without viral infection. Several food ingredients have been reported to activate pDCs. Kubo et al. showed that lactoferrin and its digestive peptides activated pDCs and induced interferon-α (IFN-α) production ex vivo [6]. Moreover, some non-pathogenic bacteria, such as lactic acid bacteria (LAB), have been reported to activate pDCs in clinical trials [7,8,9,10]. The availability and functionality of LAB are well-known. Therefore, we hypothesized that the continuous intake of LAB which activates pDCs could enhance the host’s immune system.

*Lactobacillus paragasseri* SBT2055 (LG2055) is a LAB strain that was previously classified as *Lactobacillus gasseri* and recently reclassified as *L. paragasseri* [11]. LG2055 is used for manufacturing dairy products and is known to have many additional beneficial effects, including a reduction of body fat, the prevention of abdominal adiposity [12,13,14,15], a reduction of oxidative stress [16,17], and an improvement of the intestinal environment [18]. Moreover, the immunostimulatory effects of LG2055, such as a suppression of viral infection [19,20], an induction of immunoglobulin A (IgA) production [21], and an increase in hemagglutination inhibition titers against the influenza virus after vaccination [22], have been investigated. Therefore, LG2055 is expected to maintain and/or improve the host’s immune system.

In our previous study, we examined whether LG2055 could diminish the subjective symptoms of the common cold in healthy adults using a randomized, double-blind, placebo-controlled, parallel-group comparative trial (RCT) [23]. In that study, a drinkable yogurt containing or lacking LG2055 was used as the test sample, and the ratios of the cumulative days of several local and systemic symptoms in the LG2055 group were significantly lower than those in the placebo group. Moreover, the LG2055 group showed significantly higher salivary levels of secretory IgA (sIgA) and significantly lower levels of oxidative stress markers. These results suggest that LG2055 may improve the host’s immune system and suppress the symptoms of the common cold, and imply that the ability of LG2055 to activate innate and/or acquired immunity and its antioxidative effects contributed to these results. Nevertheless, the detailed mechanisms underlying the improvement of the host immune system by LG2055 remain unclear.

Therefore, we expected that the activation of pDCs would contribute to the immunostimulatory effects of LG2055. In this study, we performed in vitro experiments to investigate whether LG2055 activates pDCs. In addition, a new RCT was performed to evaluate whether LG2055 activates pDCs and suppresses the common cold symptoms in healthy adults.

## 2. Materials and Methods

### 2.1. In Vitro Experiments

#### 2.1.1. Preparation of LG2055

LG2055 was isolated by MEGMILK SNOW BRAND and deposited in the NITE Patent Microorganisms Depositary (Chiba, Japan). LG2055 was cultivated at 37 °C in MRS broth (BD, Franklin Lakes, NJ, USA) for 16 h and harvested by centrifugation at 8000× *g* for 10 min at 4 °C. The harvested cells were washed twice with sterile saline and once with sterile distilled water. The cells were then resuspended in distilled water and lyophilized. The resulting LG2055 powder was suspended in phosphate-buffered saline (PBS) and used in the subsequent in vitro experiments.

#### 2.1.2. Flow Cytometry Analysis

Human peripheral blood mononuclear cells (PBMCs) from healthy donors were purchased from iQ Biosciences (Alameda, CA, USA) and Lonza (Basel, Switzerland). PBMCs were cultured in a RPMI 1640 medium (Thermo Fisher Scientific, Waltham, MA, USA) supplemented with 10% fetal bovine serum (Thermo Fisher Scientific), 100 U/mL penicillin, and 100 μg/mL streptomycin (Thermo Fisher Scientific) at 37 °C and 5% CO_2_.

PBMCs were seeded and treated with 10 μg/mL LG2055 for 24 h at 37 °C and 5% CO_2_. The next day, the cells were harvested and washed with PBS. The dead cells were stained with Fixable Viability Stain 780 (BD) at room temperature for 15 min. The cells were washed twice with Cell Staining Buffer (BioLegend, San Diego, CA, USA) and treated with Human TruStain Fc™ (Fc Receptor Blocking Solution) (BioLegend) in the dark for 10 min. Subsequently, the cells were stained with FITC anti-human Lineage Cocktail (CD3, CD14, CD16, CD19, CD20, CD56) (Sony, Tokyo, Japan), BV421 mouse anti-human CD123, APC mouse anti-human CD86, PE-Cy™7 mouse anti-human CD11c, and PE mouse anti-human HLA-DR (BD) antibodies on ice in the dark for 20 min. After staining, the cells were washed with a cell-staining buffer and fixed with a fixation buffer (BioLegend) for 20 min. The fixed cells were washed with the cell-staining buffer and immediately used for measurements.

Fluorescence was measured using a FACSCanto II flow cytometer (BD), and the data were analyzed using FACSDiva software version 6.1.3 (BD). In this assay, live Lineage (Lin1)− HLA-DR+ CD123+ CD11c− cells were defined as pDCs, while live Lin1− HLA-DR+ CD123− CD11c+ cells were defined as myeloid dendritic cells (mDCs), and both groups of cells were analyzed. The expression levels of cell surface markers (CD86 and HLA-DR) represented pDC and mDC activity. The mean fluorescence intensity (MFI) was measured and used for the analyses. Each experiment was performed in triplicate.

#### 2.1.3. Enzyme-Linked Immunosorbent Assay (ELISA)

PBMCs seeded in RPMI 1640 medium supplemented with 10% fetal bovine serum, 100 U/mL penicillin, and 100 μg/mL streptomycin were treated with 10 μg/mL LG2055 and 0.1 μM ssRNA (ORN R-0006; Miltenyi Biotech, Bergisch Gladbach, Germany)/DOTAP for 24 h at 37 °C and 5% CO_2_. The culture supernatants were collected by centrifugation (1500× *g*, 4 °C, 5 min) and stored at −30 °C until measurement.

The concentration of IFN-α in the supernatant was measured using an ELISA Flex: Human IFN-α (HRP) kit (MABTECH, Nacka Strand, Sweden) according to the manufacturer’s instructions. Each experiment was performed in triplicate.

#### 2.1.4. Statistical Analysis

For in vitro experiments, data were expressed as mean ± standard deviation (SD). The level of significance was determined using the Student’s *t*-test for single comparisons. A *p* value of <0.05 was considered statistically significant.

### 2.2. Clinical Study

The CONSORT checklist is shown in Supplementary Table S1.

#### 2.2.1. Participants

The participants in this study were healthy Japanese males and females aged 20–64 years who frequently caught colds. Similar to our previous trial [23], the candidates were asked a question about the frequency of catching colds during the recruitment process, and those who caught colds more often were judged to be suitable for this study. Individuals who met any of the exclusion criteria listed in Supplementary Table S2 were excluded. The study participants were instructed to follow the instructions listed in Supplementary Table S3 throughout the study period.

All participants had the capacity to provide consent and applied for the study of their own will after sufficiently understanding the study. The purpose and details of this study were sufficiently explained to the participants, and written informed consent was obtained from all participants before initiation of the study.

#### 2.2.2. Test Samples

We prepared capsules containing two types of test samples: an active sample containing LG2055 and the placebo that did not contain LG2055. The test samples were manufactured by API Co., Ltd. (Gifu, Japan). To prepare the active capsules, materials such as LG2055 powder and starch were mixed and filled into hard capsules. Subsequently, the capsules were enteric-coated and used as active capsules. The active capsules contained more than 1 × 10^9^ colony-forming units (cfu) of LG2055/3 grains. The placebo capsules were prepared in the same manner, except that the LG2055 powder was substituted with starch. All test samples were indistinguishable in color, shape, or taste. The nutritional composition of each test sample is shown in Table 1. In this study, the participants ingested three active or placebo capsules once a day for 12 weeks.

#### 2.2.3. Study Design

The study design was based on our previous study [23]. This clinical trial was designed as an RCT. This study protocol received the approval of the Institutional Review Board of the Ethics Committee of Nihonbashi Cardiology Clinic (Tokyo, Japan) on 28 September 2022 (approval number: NJI-022-09-01) and was registered in the University Hospital Medical Information Network Clinical Trials Registry prior to its initiation (UMIN ID: UMIN000049183). This study was conducted from October 2022 to May 2023 in accordance with the principles of the Helsinki Declaration of 1975 (revised 2013), and the Ethical Guidelines for Medical and Health Research Involving Human Subjects issued by the Ministry of Education, Culture, Sports, Science and Technology and the Ministry of Health, Labour and Welfare of Japan.

A screening test, a pre-observation period of 2 weeks, and an intake period of 12 weeks were included in this study. The participants were instructed to answer a physical health questionnaire every day and received a medical doctor’s consultation four times during the study (during the screening test and at 0, 6, and 12 weeks during the intake period).

#### 2.2.4. Outcomes

The primary outcome of this study was the result of the daily physical health questionnaire survey on the common cold symptoms. The secondary outcomes were the salivary sIgA concentration, the secretion rate, and the amount of secretion; serum IgA and immunoglobulin G (IgG) levels; NK cell activity; pDC activity; body temperature; fecal microbiota, and the Profile of Mood States, 2nd Edition (POMS2) score.

#### 2.2.5. Physical Health Questionnaire

We used a daily physical health questionnaire designed in our previous clinical trial [23]. The questionnaire was designed in accordance with the Wisconsin Upper Respiratory Symptom Survey (WURSS)-21, WURSS-24 [24], and other previous studies [25,26]. Subjective local symptoms (runny nose, plugged nose, sneezing, sore throat, hoarseness, cough, and headache) and systemic symptoms (feeling tired, chill, and fever) were evaluated in five grades (No symptoms, Very mild, Mild, Moderate, and Severe) using a daily physical health questionnaire. This survey was conducted in the Japanese language. In addition to evaluating individual symptoms, the participants’ comprehensive condition was evaluated using a face scale (five grades; “feeling clear” to “feeling like crying”) at the same time.

#### 2.2.6. Salivary sIgA, Serum IgA, Serum IgG, and NK Cell Activity

Saliva and blood samples were collected during the screening test and at 0, 6, and 12 weeks after the start of intake. Sample collection and the measurement of each parameter were conducted according to the methods described in our previous study [23]. NK cell activity was not measured during the screening.

#### 2.2.7. pDC Activity

Blood samples were collected at 0, 6, and 12 weeks after the start of intake. PBMCs were obtained from blood samples, frozen, and stored until measurement. The frozen PBMCs were thawed, and live cells were counted by trypan blue staining. A total of 5 × 10^5^ live cells were suspended with Stain Buffer (BD) and treated with BD Fc Block™ Reagent for Human (BD) at 4 °C for 10 min. Subsequently, fluorescent dye-conjugated antibodies (FITC anti-human CD123, APC anti-human CD304 [Neuropilin-1; BioLegend], PE anti-human CD86 [Thermo Fisher Scientific], PerCP-Cy™5.5 mouse anti-human HLA-DR [BD]) were added and the cells were stained at 4 °C for 30 min in the dark. Stained cells were fixed with 4% paraformaldehyde/PBS and measured using Accuri C6 flow cytometer (BD). CD123+ CD304+ cells were gated as pDCs, and the expression levels of cell surface markers (CD86 and HLA-DR) were used to measure pDC activity.

#### 2.2.8. Analysis of Fecal Microbiota

Fecal samples were collected by participants themselves using a collection kit (Kyoto Institute of Nutrition & Pathology, Inc., Kyoto, Japan) and suspended in DESS buffer (0.25 M disodium ethylenediaminetetraacetic acid pH 8.0, 20% dimethyl sulfoxide, NaCl saturated) [27] at 0 and 12 weeks after the start of intake. Collected fecal samples were stored at 4 °C and submitted within five days after collection. The samples were centrifuged at 15,000× *g* for 5 min at room temperature, and bacterial DNA was extracted using the QuickGene-810 system (Bio Medical Science, Tokyo, Japan) and QuickGene DNA tissue kit (KURABO, Osaka, Japan), as reported previously [28].

The V3 and V4 regions of the 16S rRNA gene amplicons were prepared according to the 16S Metagenomic Sequencing Library Preparation (Illumina, San Diego, CA, USA), and sequencing was conducted using the Illumina Miseq system (Illumina). After sequencing, the obtained fastq files were analyzed using Quantitative Insights into Microbial Ecology 2 (QIIME2). The SILVA database (ver. 138) was used for taxonomic classification. The relative abundance (genus level) was calculated, and analyzed for genera that showed more than 0.01% mean relative abundance. The genus *Lactobacillus* was reclassified into 25 genera in 2020 [29]; however, the SILVA database (ver. 138) did not support reclassification. Therefore, we performed the analysis according to the old classification.

Moreover, k-means clustering based on the composition of the fecal microbiota (0 week) was performed, and the fecal microbiome of participants was divided into three clusters (k = 3). We used EZR software version 1.40 to perform k-means clustering. The initial seed value was set to 10. The genera detected frequently (more than 50% of the samples) were selected and used for the clustering. In addition, the genera not selected were classified together as “other genera”.

#### 2.2.9. Safety Assessments

For safety assessments, participants received a doctor’s consultation, and subjective and objective symptoms were determined in a screening test and at 0, 6, and 12 weeks after the start of intake. Body weight (body mass index), blood pressure, and pulse were measured simultaneously. Hematological parameters (hemoglobin, hematocrit, white and red blood cell counts, and blood platelets), biochemical parameters (total protein, albumin, total bilirubin, aspartate aminotransferase, alanine transaminase, lactate dehydrogenase, alkaline phosphatase, gamma-glutamyl transpeptidase, glucose, hemoglobin A1c, sodium, potassium, chloride, triglycerides, total cholesterol, high-density lipoprotein cholesterol, low-density lipoprotein cholesterol, blood urea nitrogen, and uric acid), and urinalysis parameters (urobilinogen [qualitative], bilirubin [qualitative], protein [qualitative], glucose [qualitative], ketones, occult blood, and pH) in the participants’ blood and urine samples were measured in the screening tests and at 0 and 12 weeks after starting intake. These measurements were performed by LSI Medience Corporation (Tokyo, Japan).

The participants were instructed to maintain a daily record during the pre-observation and intake periods. The records covered defecation, diarrhea, menstruation, medication use, outings, test sample consumption, and physical condition.

#### 2.2.10. Sample Size

The sample size was determined on the basis of the data derived from our previous study [23]. On the basis of the data for cumulative days of total subjective symptoms in the previous study, more than 84 participants in each group were necessary to detect intergroup differences at a 5% significance level and a statistical power of 80%. Therefore, 100 participants were included in each group considering a dropout rate of 10%.

#### 2.2.11. Randomization

On the basis of sex, age, salivary sIgA level, serum IgA level, serum IgG level, POMS2 score, COVID-19 vaccination, and influenza vaccination status, the participants were divided into two groups according to the block-randomization method. Subsequently, an independent controller assigned each group to either the LG2055 or placebo group. The controller sealed the assignment list until the designated unmasking time.

#### 2.2.12. Statistical Analysis

In the clinical study, the cumulative days of each symptom during the intake period was calculated on the basis of the physical health questionnaire survey and analyzed using the chi-square test for the primary outcome.

On the basis of the measured values, averages and SD values were calculated for each immunological marker. The normal distribution of each data point was evaluated using the Shapiro–Wilk test. For variables showing a normal distribution, between-group comparisons were performed using an unpaired *t*-test. For variables showing a non-normal distribution, the Wilcoxon rank-sum test was used for between-group comparisons.

In the fecal microbiota analysis, between-group comparisons were performed using the Wilcoxon rank-sum test, and within-group comparisons between 0 and 12 weeks were performed using the Wilcoxon signed-rank test.

Statistical analyses were performed using the IBM SPSS Statistics version 27. A *p* value of <0.05 was considered statistically significant.

## 3. Results

### 3.1. In Vitro Experiments

**Figure 1:**
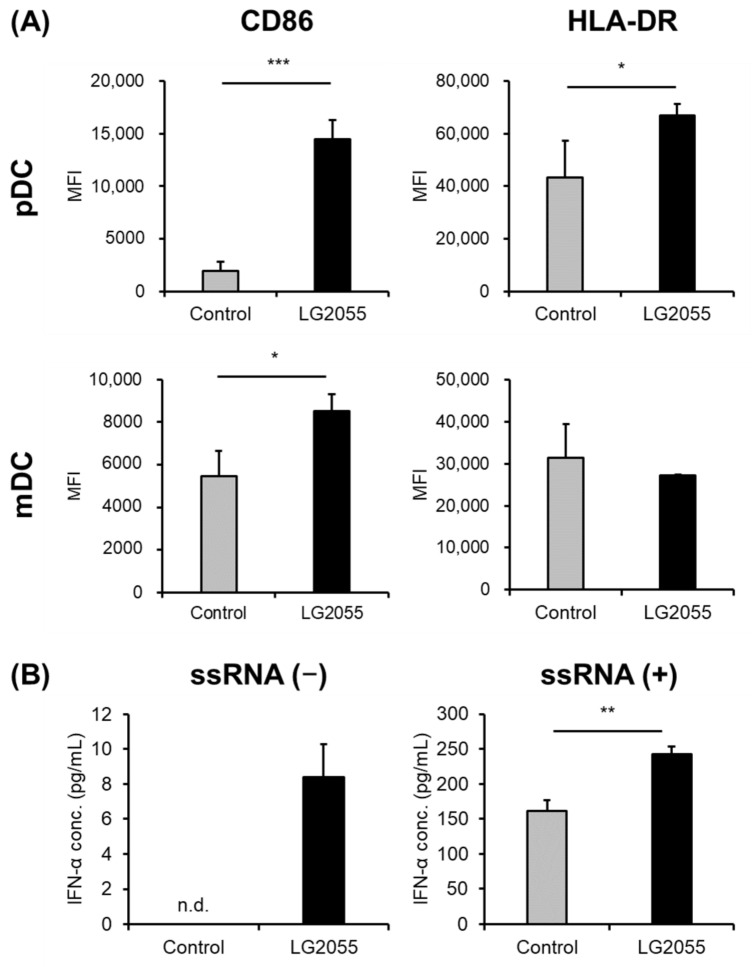
Evaluation of pDC and mDC activation by LG2055 in vitro. (**A**) PBMCs were treated with LG2055 for 24 h, and CD86 and HLA-DR expressions on pDCs and mDCs were evaluated by flow cytometry. (**B**) PBMCs were treated with LG2055 and ssRNA or LG2055 only for 24 h and each culture supernatant was collected. IFN-α concentration in the supernatant was measured by ELISA. (**A**,**B**) Each experiment was performed in triplicate; data are shown as mean ± SD. * *p* < 0.05, ** *p* < 0.01, *** *p* < 0.001 according to the Student’s *t*-test. n.d., not detected.

To confirm that LG2055 activates dendritic cells, we performed in vitro assays using PBMCs. PBMCs from four different donors were treated with LG2055, and the expression levels of CD86 and HLA-DR were evaluated to determine pDC and mDC activity. Increased expression of CD86 and HLA-DR was observed in three donors, whereas no increase in expression was observed in the fourth donor (Figure 1A, Supplementary Figure S1). We also evaluated mDC activity, but no obvious changes were observed in all four donors (Figure 1A, Supplementary Figure S2). Subsequently, the IFN-α concentration in PBMC culture supernatants was measured and a high IFN-α concentration was observed in LG2055-treated PBMCs. In addition, LG2055 significantly increased IFN-α concentration in the supernatant of cells stimulated with ssRNA (Figure 1B). Since pDCs are the main source of IFN-α in PBMCs and are the only cells within PBMCs that produce IFN-α in response to CpG ODN [30,31], LG2055 is thought to induce IFN-α production in PBMCs via pDC activation. Activated pDCs produce large amounts of type I interferons, such as IFN-α, and contribute to the protection against viral infections [5]. Therefore, our results suggest that LG2055 activates pDCs and induces IFN-α production.

### 3.2. Clinical Study

#### 3.2.1. Participants

**Figure 2:**
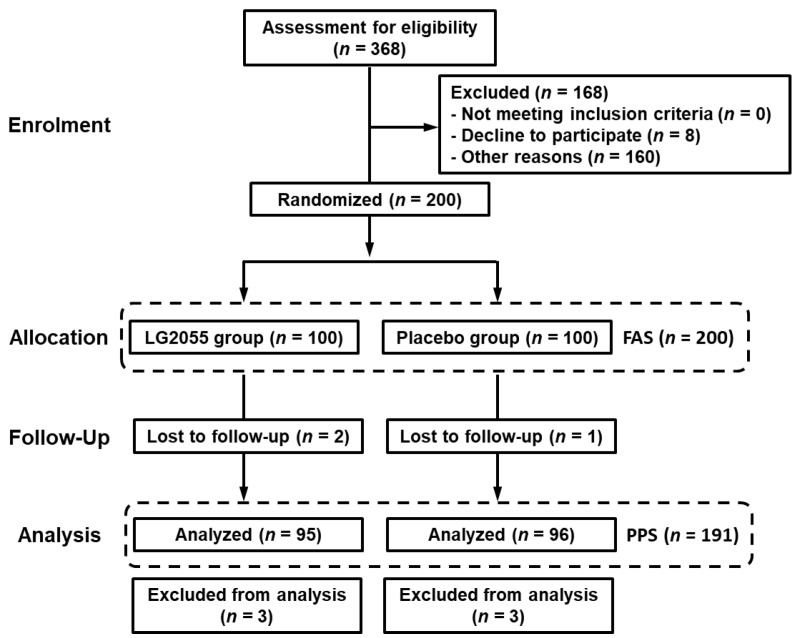
Flow diagram of this study.

Figure 2 shows a flowchart of the participant-selection process. Participant recruitment for this study began in September 2022. From 368 candidates, a total of 200 participants were considered appropriate for this study and were enrolled. They were randomly divided into two groups (LG2055 and placebo groups) according to the allocation factors and were administered the corresponding test sample. During the intake period, three participants discontinued the trial because of relocation, long-term overseas business trips, and arrhythmia development, respectively. Thus, 197 participants completed the scheduled intake of the test samples. Six participants were excluded because they were taking medications that could have influenced their immune system during the intake period. Consequently, 191 participants (95 in the LG2055 group and 96 in the placebo group) in the per-protocol set (PPS) were included in the efficacy analysis.

#### 3.2.2. Background of Participants

Table 2 presents the participants’ background characteristics. The two groups showed no significant differences for any of the background characteristics. In each group, the levels of hematological and biochemical markers were within normal ranges.

#### 3.2.3. Intake Ratio

The intake ratio of the test samples was over 97.6% among the 197 participants who completed the scheduled intake of the test samples. The average intake ratios were 99.95% in the LG2055 group and 99.96% in the placebo group. The two groups showed no statistically significant differences in the intake ratios.

#### 3.2.4. Primary Outcome

To evaluate the incidence ratio, the severity of each symptom in the results of the daily physical questionnaire survey was converted into two grades according to the previous study [23], and the cumulative days of each symptom was calculated. Thus, “No symptoms” was converted to “without symptoms”, and “Very mild”, “Mild”, “Moderate”, and “Severe” were together categorized as “with symptoms”. The data for the cumulative days of each symptom are shown in Table 3. The LG2055 group showed a significantly higher ratio of “without symptoms” for runny nose, plugged nose, sneezing, sore throat, hoarseness, and chill in comparison with the placebo group. On the other hand, the LG2055 group showed a significantly lower ratio of “without symptoms” for headache and feeling tired. The rates of cough and fever did not differ significantly between the groups.

In the evaluation of the comprehensive condition using a face scale, the results were categorized as “good” (1 grade) and “bad” (4 grades), and the cumulative days were calculated (Table 4). The LG2055 group showed a significantly higher ratio of “good” results during the intake period than the placebo group.

Overall, the results of the daily physical questionnaire survey indicated that the incidence rates of symptoms associated with the common cold were lower in the LG2055 group.

#### 3.2.5. pDC Activity

**Figure 3:**
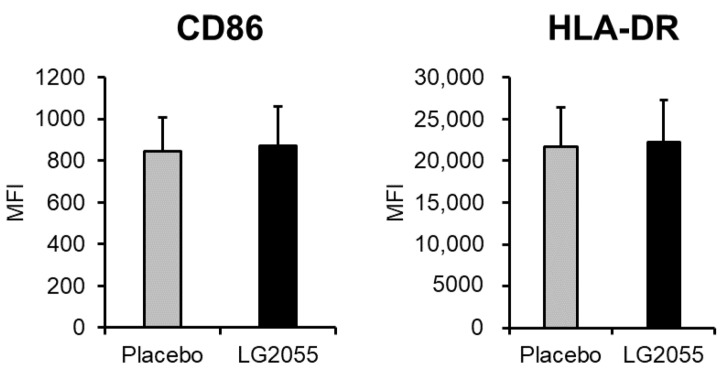
pDC activity (CD86 and HLA-DR expression) at 12 weeks. PBMCs obtained from blood samples were stained and analyzed by flow cytometry. CD86 and HLA-DR expression on pDCs were evaluated as pDC activity. Data are shown as mean ± SD (LG2055 group *n* = 95; placebo group *n* = 96).

In the LG2055 group, the MFI values of CD86 and HLA-DR were higher than those in the placebo group at 6 and 12 weeks after the start of intake; however, no significant difference was observed between the two groups (Figure 3, Supplementary Table S4).

#### 3.2.6. Salivary sIgA, Serum IgA, Serum IgG Levels, and NK Cell Activity

The LG2055 and placebo groups showed no significant differences in the secretion rate of salivary sIgA, serum IgA levels, serum IgG levels, or NK cell activity (Supplementary Table S5).

#### 3.2.7. Analysis of Fecal Microbiota

The percentage of genus *Lactobacillus* in the LG2055 group was significantly higher than that in the placebo group at 12 weeks (0.03% ± 0.10% vs. 0.02% ± 0.15%, *p* < 0.01). In addition, within-group comparisons showed that the percentages of the genera *Butyricimonas* and *Agathobacter*, which were butyric acid-producing bacteria, at 12 weeks were significantly greater than those at 0 week in the LG2055 group. The percentages of *Butyricimonas* and *Agathobacter* did not differ significantly between the two groups (Supplementary Table S6).

#### 3.2.8. Stratified Analysis

A stratified analysis was performed to reveal the relationship between the condition of the immune system and the effects of LG2055. It was expected that the immunostimulatory effects of LG2055 may be observable especially in immune-weakened participants. The salivary sIgA secretion rate at 0 week was used to subdivide the participants. The 112 participants (57 in the LG2055 group and 55 in the Placebo group) who showed salivary sIgA secretion rates under the PPS average (110.3 μg/min) were regarded as immune-weakened participants and selected for the stratified analysis. No significant between-group differences were observed in the background characteristics of these participants.

**Figure 4:**
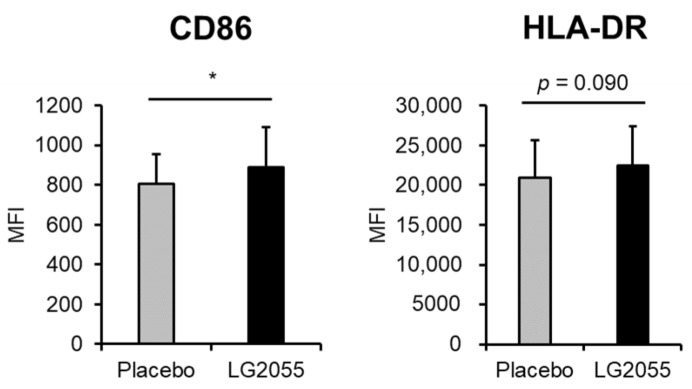
pDC activity (CD86 and HLA-DR expression) at 12 weeks (stratified analysis). PBMCs obtained from blood samples were stained and analyzed by flow cytometry. CD86 and HLA-DR expression on pDCs were evaluated as pDC activity. Participants who showed salivary sIgA secretion rates under the PPS average were selected for the stratified analysis (LG2055 group *n* = 57; Placebo group *n* = 55). Data are shown as mean ± SD. * *p* < 0.05.

In comparison with the placebo group, CD86 expression was significantly higher in the LG2055 group at 12 weeks. In addition, HLA-DR expression in the LG2055 group tended to be higher than that in the placebo group at 12 weeks (Figure 4, Supplementary Table S7). Among participants who showed high secretion rates (more than 110.3 μg/min) of salivary sIgA (38 in the LG2055 group and 41 in the placebo group), CD86 and HLA-DR expressions showed no significant differences between the two groups. These results indicated that pDCs were activated in the LG2055 group at 12 weeks, suggesting that LG2055 intake activated pDCs in the participants with low salivary sIgA secretion rates.

Moreover, the daily physical questionnaire survey results indicated that the LG2055 group included a significantly higher ratio of “without symptoms” for runny nose, plugged nose, sneezing, hoarseness, and chill than the placebo group in this stratified analysis (Table 5). The incidence of headache was higher in the LG2055 group than in the placebo group. In evaluations performed using the face scale, the LG2055 group showed a significantly higher ratio of “good” responses than the placebo group during the intake period (Table 6). These results of the stratified analysis indicated that the subjective symptoms of the common cold were suppressed in the LG2055 group.

#### 3.2.9. Safety Assessment

In this study, 55 adverse events were reported by 32 participants. All adverse events were judged by the medical doctor not to be, or probably not to be, associate with the intake of the test samples. Thus, we concluded that the intake of the test samples caused no adverse events.

## 4. Discussion

In the previous study, we reported that the intake of yogurt containing LG2055 suppressed the subjective symptoms of the common cold in healthy adults [23]. In addition, the upregulation of salivary sIgA levels and the suppression of oxidative stress induced by LG2055 intake have been suggested to contribute to the suppression of symptoms. However, the underlying mechanisms of action of LG2055 have not been sufficiently elucidated. Therefore, we focused on pDCs, which play important roles in the host’s defense against viral infections. Viral infection is the major cause of the common cold, therefore, we investigated whether the activation of pDCs by LG2055 contributes to the suppression of the subjective symptoms of the common cold. We investigated whether LG2055 could activate pDCs in vitro. The expression of CD86 and HLA-DR on pDCs was significantly upregulated by the treatment with LG2055, and an enhanced type I interferon (IFN-α) production was observed in LG2055-treated PBMCs (Figure 1), indicating that LG2055 activated pDCs.

Next, we performed a clinical trial and found that the LG2055 group showed significantly lower ratios of cumulative days of several local and systemic symptoms than the placebo group (Table 3), consistent with our previous study, but the findings showed no significant difference in pDC activity (expression levels of CD86 and HLA-DR) between the LG2055 and placebo groups (Figure 3, Supplementary Table S4). Subsequently, we performed a stratified analysis intended for immune-weakened participants and found that LG2055 intake enhanced the expression of CD86 and HLA-DR on pDCs in participants who showed low salivary sIgA secretion rates (Figure 4, Supplementary Table S7). Furthermore, LG2055 was shown to suppress the subjective symptoms of the common cold in the stratified analysis (Table 5). These findings indicate that LG2055 contributed to the maintenance of the physical condition by activating pDCs and improving the host’s immune system.

In this study, the secretion rate of salivary sIgA was used to select immune-weakened participants. Salivary sIgA is known to bind to various pathogenic organisms and prevent host infections [32]. The salivary sIgA secretion level may reflect the immune function in the oral cavity and upper respiratory tract. Moreover, salivary sIgA has been suggested to be a potential measure of mucosal and systemic immunity [33]. Decreased salivary sIgA secretion increases the risk of common cold symptoms [34,35]. Additionally, pDCs have been reported to play an important role in sIgA induction [36]. On the basis of these reports, the use of salivary sIgA values to select immune-weakened participants was considered appropriate.

Sugimura et al. reported that *Lactococcus lactis* JCM 5805, one of LAB, activates pDCs in healthy adults, and that *L. lactis* JCM 5805 intake suppresses the risk of morbidity from the common cold [37]. In their report, participants were subdivided into two layers according to the level of HLA-DR expression, and the rate of change in HLA-DR expression in the *L. lactis* JCM 5805 group was significantly higher than that in the placebo group in the low pDC activity layer, but not in the high pDC activity layer. These findings indicate that the participants with low pDC activity, who were regarded as immune-weakened, showed a greater predisposition to the stimulatory effects of *L. lactis* JCM 5805. These data support our results showing that LG2055 activates pDCs, especially in immune-weakened participants.

Activated pDCs are known to produce type I interferon, activate various immune cells, such as NK, B, and T cells, and strengthen the host’s immune system, thus, activated pDCs are considered to contribute to the prevention of infection [5]. In addition, some studies have investigated the influence of LAB intake on common cold symptoms and have suggested that several strains of LAB activate pDCs and ameliorate common cold symptoms [10,25,26,37]. Many previous studies, including clinical studies and in vivo studies, have reported that LG2055 upregulates antiviral factors [19,20], induces IgA production [21], enhances NK cell activity [22], and increases hemagglutination inhibition titers against the influenza virus after vaccination [22]. These immunostimulatory effects of LG2055 could be explained by pDC activation and the subsequent activation of other immune cells. It is supposed that LG2055 intake activates pDCs and other immune cells, maintains an appropriate host immune system, and prevents the incidence of common cold symptoms.

pDCs have been suggested to take up LAB and recognize bacterial DNA via TLR9 [37]. On the other hand, human pDCs have been reported to recognize gram-positive bacteria via TLR1/2 [38]. In a previous report, LG2055 was shown to enhance IgA production via TLR2 [21], thus TLR2 may contribute to pDC activation by LG2055. The mechanisms by which LG2055 activates pDCs have not been elucidated, and various pathways have been proposed. Further studies are required to elucidate the underlying mechanisms of pDC activation by LG2055.

We used a physical health questionnaire survey to assess the primary outcome of this study. As expected, the incidence of many symptoms was reduced in the LG2055 group (Table 3). However, the opposite results were obtained for headache and feeling tired. This finding was assumed to be attributable to the influence of chronic symptoms. Headache and feeling tired are common acute symptoms of the common cold, however, chronic headache and feeling tired are also frequently observed for other reasons, e.g., as a result of migraines [39] or meteoropathy [40,41]. Therefore, the evaluations of headache and feeling tired can be considered to be more easily influenced by chronic symptoms rather than other common cold symptoms. Thus, the results obtained for headache and feeling tired in this survey may have been influenced by chronic symptoms, and assessments of headache and feeling tired in relation to the symptoms caused by the common cold should be done carefully. In addition, among studies reporting the efficiency of food ingredients for common cold symptoms using a similar method, some examples have shown opposite results for several symptoms [42,43]. Although questionnaire surveys are useful for evaluating common cold symptoms, it should be noted that the subjectivity of participants cannot be excluded. Therefore, comprehensive consideration is important to determine whether a functional ingredient is effective in the suppression of common cold symptoms. In this study, since LG2055 suppressed the incidence of most symptoms, we concluded that LG2055 intake contributes to a decrease in the subjective symptoms of the common cold.

LG2055 has been reported to act as a probiotic and to improve the intestinal environment [18,44,45], potentially increasing the levels of bacteria or metabolites with immunostimulatory effects in the host’s gut. Therefore, we performed a fecal microbiota analysis and calculated the ratio of each genus. In comparison with the placebo group, the percentage of the genus *Lactobacillus* was significantly higher in the LG2055 group at 12 weeks, as expected (Supplementary Table S6). Although identification was performed only at the genus level, the increase in the percentage of *Lactobacillus* can be expected to have been mainly induced by LG2055 intake. In addition, within-group comparisons revealed that LG2055 intake increased the percentages of the genera *Butyricimonas* and *Agathobacter* (Supplementary Table S6), which are butyrate-producing bacteria. Butyrate, one of short-chain fatty acids (SCFAs), has been reported to show various functions related to the immune system [46]. Furthermore, SCFAs have been suggested to be associated with IgA production [47]. In a previous report, an increased molar ratio of butyrate to total SCFA was observed in the cecum of LG2055-fed rats [48]. Thus, LG2055 may increase butyrate production in the intestine and contribute to the maintenance of the host’s immune system.

On the basis of the bacteriological composition, the human gut microbiota can be classified into several groups (enterotypes). Enterotypes are traditionally categorized as *Bacteroides* type, *Prevotella* type, and *Ruminococcus* type [49]. Subsequently, several enterotype classifications have been proposed [50,51]. The gut microbiota of the Japanese population has been recently shown to differ from those of other populations [52], thus, the classification of enterotypes may differ in relation to background characteristics such as race or region. The composition of the gut microbiota is linked to the host’s health and disease. Additionally, the gut microbiota is related to the immune system [53]. Therefore, we performed an exploratory analysis to investigate whether the pDC-activating effects of LG2055 were affected by differences in the gut microbiota. K-means clustering of the gut microbiota composition at 0 week divided the 191 participants into three clusters. The percentage of genus *Prevotella* was the highest in Cluster 1, and that of genus *Bacteroides* was the highest in Cluster 2 (Supplementary Table S8). pDC activation by LG2055 was evaluated in each cluster, and a significant upregulation of CD86 expression in the LG2055 group was observed in Cluster 1 at 12 weeks. In Cluster 3, the CD86 expression in the LG2055 group at 12 weeks was higher than that in the placebo group. In contrast, no significant difference in CD86 expression was observed between the LG2055 and placebo groups in Cluster 2 (Supplementary Table S9). These results suggested that the pDC-activating effect of LG2055 may depend on the host’s gut microbiota. Substances such as metabolites produced by gut bacteria (e.g., SCFAs) and bacterial components are known to stimulate the host’s immune cells and play important roles in the host’s immune system [53,54]. Differences in the host’s gut microbiota have been supposed to cause differences in stimuli at a steady state and may affect the expression of the effects of LG2055. For example, SCFA production from dietary fibers has been reported to differ between *Prevotella*-dominant and *Bacteroides*-dominant microbiota [55]. The relationship between the specific differences in the host’s gut microbiota and pDC activation is expected to be revealed in the future.

## 5. Conclusions

In conclusion, we performed in vitro experiments and an RCT to evaluate whether LG2055 suppresses common cold symptoms by activating pDCs in healthy adults. Our results showed that LG2055 activated pDCs in vitro, and that a daily intake of LG2055 decreased the cumulative days of common cold symptoms in healthy adults. Furthermore, the expression of CD86 and HLA-DR in the LG2055 group was higher than that in the placebo group among the participants with a low secretory rate of salivary sIgA. These results suggest that LG2055 intake suppresses the subjective symptoms of the common cold by activating pDCs and improving the host’s immune system in healthy adults, especially in immune-weakened adults.

## Figures and Tables

**Table 1:** Nutritional composition of the test samples (per 3 grains).

		Active Capsules	Placebo Capsules
Energy	kcal	3.6	3.7
Protein	g	0.06	0.01
Fat	g	0.03	0.03
Sugar	g	0.8	0.9
Sodium	mg	2.2	0.3
LG2055	cfu	>1 × 10^9^	not detected

**Table 2:** Background characteristics of the participants.

		LG2055 Group	Placebo Group	*p*-Value
		*n* = 100	*n* = 100	
Sex	male/female	52/48	55/45	0.671
Age	years	47.0 ± 9.4	47.4 ± 8.9	0.758
Height	cm	165.4 ± 7.0	165.0 ± 8.7	0.775
Weight	kg	61.0 ± 10.4	61.3 ± 11.4	0.815
BMI	kg/m^2^	22.2 ± 2.9	22.4 ± 3.2	0.614

Values are presented as mean ± SD. BMI, body mass index.

**Table 3:** Comparison of the cumulative days of each symptom (two grades).

Symptoms	Group	*n*	With Symptoms		Without Symptoms		*p*-Value
			Cumulative Days	Ratio	Cumulative Days	Ratio	
Runny nose	LG2055	7981	2674	33.5%	5307	66.5%	<0.001 *
	Placebo	8065	2968	36.8%	5097	63.2%	
Plugged nose	LG2055	7981	1793	22.5%	6188	77.5%	0.005 *
	Placebo	8065	1965	24.4%	6100	75.6%	
Sneezing	LG2055	7981	1539	19.3%	6442	80.7%	<0.001 *
	Placebo	8065	2067	25.6%	5998	74.4%	
Sore throat	LG2055	7981	652	8.2%	7329	91.8%	0.002 *
	Placebo	8065	769	9.5%	7296	90.5%	
Hoarseness	LG2055	7981	244	3.1%	7737	96.9%	<0.001 *
	Placebo	8065	516	6.4%	7549	93.6%	
Cough	LG2055	7981	963	12.1%	7018	87.9%	0.964
	Placebo	8065	975	12.1%	7090	87.9%	
Headache	LG2055	7981	969	12.1%	7012	87.9%	<0.001 *
	Placebo	8065	755	9.4%	7310	90.6%	
Feeling tired	LG2055	7981	1483	18.6%	6498	81.4%	<0.001 *
	Placebo	8065	1271	15.8%	6794	84.2%	
Chill	LG2055	7981	216	2.7%	7765	97.3%	<0.001 *
	Placebo	8065	400	5.0%	7665	95.0%	
Fever	LG2055	7981	103	1.3%	7878	98.7%	0.244
	Placebo	8065	88	1.1%	7977	98.9%	

* Significant difference between the two groups (*p* < 0.05).

**Table 4:** Comparison of the cumulative days on the face scale (two grades).

Group	*n*	Bad		Good		*p*-Value
		Cumulative Days	Ratio	Cumulative Days	Ratio	
LG2055	7981	4789	60.0%	3192	40.0%	<0.001 *
Placebo	8065	5145	63.8%	2920	36.2%	

* Significant difference between the two groups (*p* < 0.05).

**Table 5:** Comparison of the cumulative days of each symptom (two grades) (stratified analysis).

Symptoms	Group	*n*	With Symptoms		Without Symptoms		*p*-Value
			Cumulative Days	Ratio	Cumulative Days	Ratio	
Runny nose	LG2055	4789	1701	35.5%	3088	64.5%	<0.001 *
	Placebo	4621	2020	43.7%	2601	56.3%	
Plugged nose	LG2055	4789	1078	22.5%	3711	77.5%	<0.001 *
	Placebo	4621	1302	28.2%	3319	71.8%	
Sneezing	LG2055	4789	940	19.6%	3849	80.4%	<0.001 *
	Placebo	4621	1341	29.0%	3280	71.0%	
Sore throat	LG2055	4789	405	8.5%	4384	91.5%	0.427
	Placebo	4621	370	8.0%	4251	92.0%	
Hoarseness	LG2055	4789	165	3.4%	4624	96.6%	<0.001 *
	Placebo	4621	246	5.3%	4375	94.7%	
Cough	LG2055	4789	637	13.3%	4152	86.7%	0.307
	Placebo	4621	582	12.6%	4039	87.4%	
Headache	LG2055	4789	595	12.4%	4194	87.6%	<0.001 *
	Placebo	4621	425	9.2%	4196	90.8%	
Feeling tired	LG2055	4789	743	15.5%	4046	84.5%	0.314
	Placebo	4621	752	16.3%	3869	83.7%	
Chill	LG2055	4789	86	1.8%	4703	98.2%	<0.001 *
	Placebo	4621	252	5.5%	4369	94.5%	
Fever	LG2055	4789	60	1.3%	4729	98.7%	0.503
	Placebo	4621	51	1.1%	4570	98.9%	

* Significant difference between the two groups (*p* < 0.05).

**Table 6:** Comparison of the cumulative days of face scale (two grades) (stratified analysis).

Group	*n*	Bad		Good		*p*-Value
		Cumulative Days	Ratio	Cumulative Days	Ratio	
LG2055	4789	2779	58.0%	2010	42.0%	<0.001 *
Placebo	4621	2915	63.1%	1706	36.9%	

* Significant difference between the two groups (*p* < 0.05).

## Data Availability

The datasets used and/or analyzed in the current study are available from the corresponding author upon request.

## Supplementary Materials

The following supporting information can be downloaded at: https://www.mdpi.com/article/10.3390/nu15204458/s1, Figure S1. Evaluation of pDC activation by LG2055 in PBMCs from different donors; Figure S2. Evaluation of mDC activation by LG2055 in PBMCs from different donors; Table S1. CONSORT 2010 checklist of information in this report; Table S2. Exclusion criteria; Table S3. Instructions followed by the participants during the study; Table S4. Comparison of pDC activity (CD86 and HLA-DR expression); Table S5. Comparison of immunological markers; Table S6. Effects of LG2055 intake on fecal microbiota; Table S7 Comparison of pDC activity (CD86 and HLA-DR expression) (stratified analysis); Table S8. Bacterial composition of each cluster (top 10 genera); Table S9. Comparison of pDC activity (CD86 expression) in each cluster.
