# Supplementary material for: Lactobacillus paragasseri SBT2055 Activates Plasmacytoid Dendritic Cells and Improves Subjective Symptoms of Common Cold in Healthy Adults: A Randomized, Double-Blind, Placebo-Controlled Parallel-Group Comparative Trial
Source: Nutrients. 2023 Oct 20;15(20):4458. doi: 10.3390/nu15204458 (PMC10610513; doi:10.3390/nu15204458)
Supplement: Supplementary file 1 [file nutrients-15-04458-s001.zip › nutrients-2628079-supplementary.pdf]

Supplementary materials

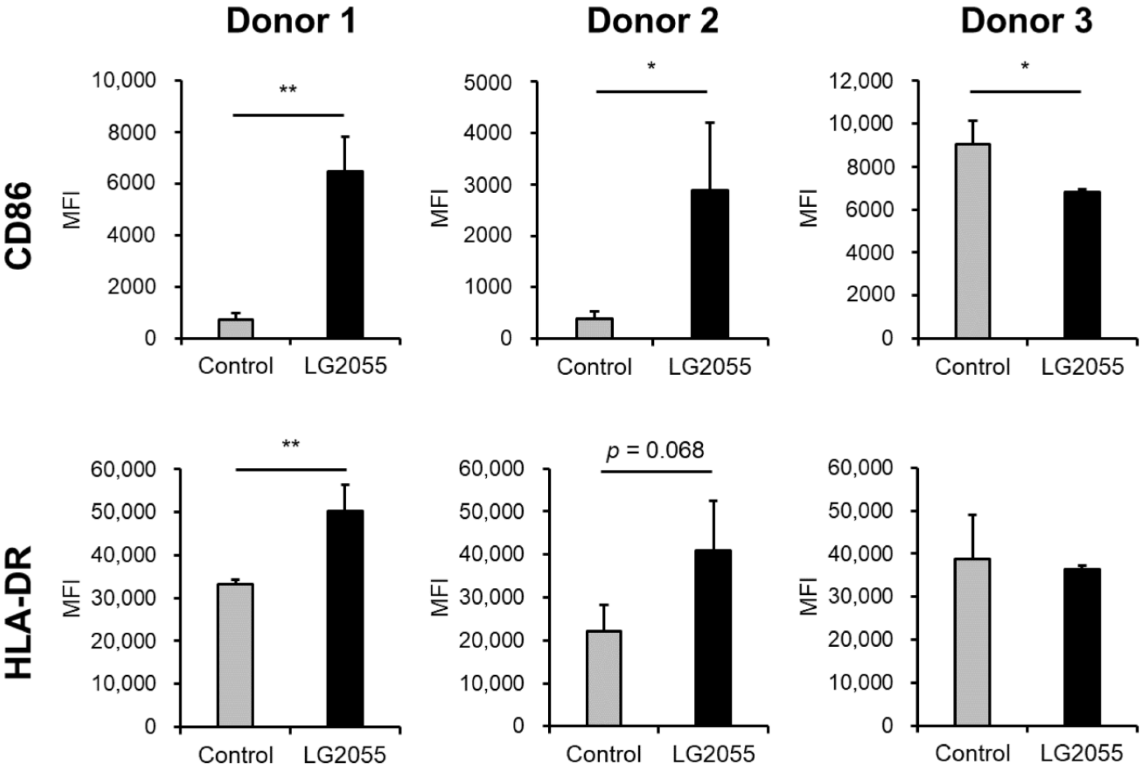

Supplementary Figure S1. Evaluation of pDC activation by LG2055 in PBMCs from different donors. PBMCs were treated with LG2055 for 24 h and analyzed CD86 and HLA-DR expression on pDCs by flow cytometry. Each experiment was performed in triplicate; data are shown as mean ± SD. * $p < 0.05$, ** $p < 0.01$ according to the Student's *t*-test.

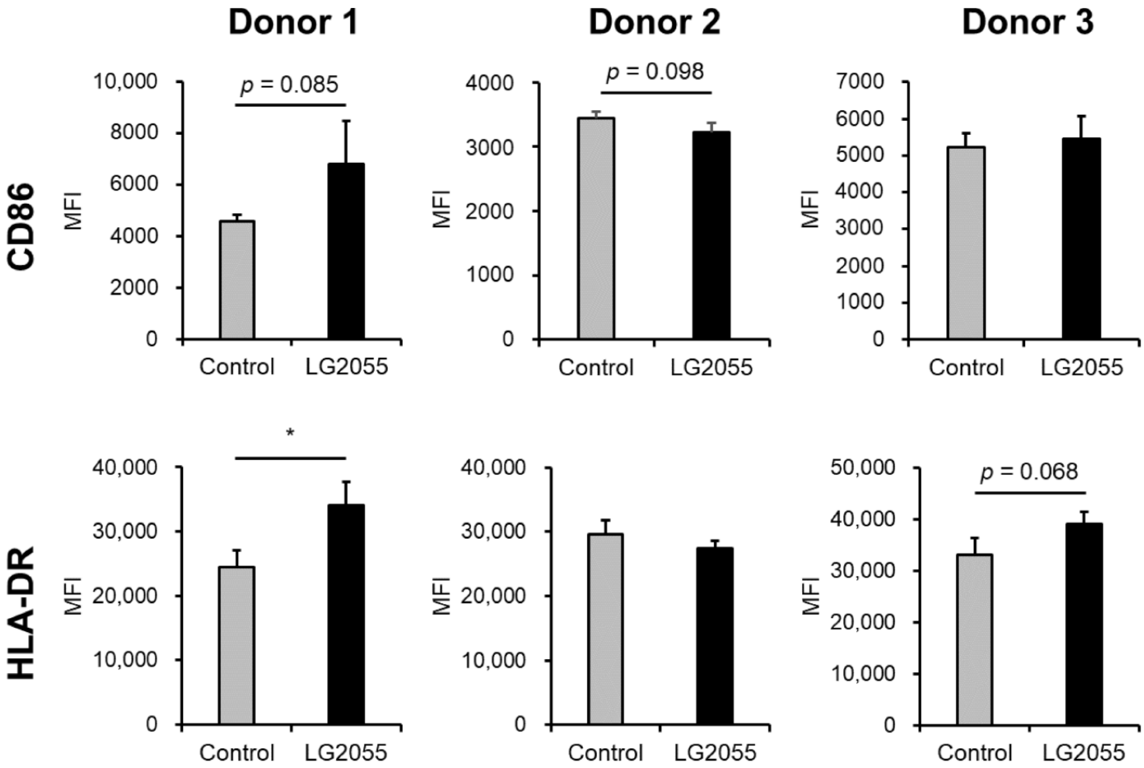

Supplementary Figure S2. Evaluation of mDC activation by LG2055 in PBMCs from different donors. PBMCs were treated with LG2055 for 24 h and analyzed CD86 and HLA-DR expression on mDCs by flow cytometry. Each experiment was performed in triplicate; data are shown as mean ± SD. * $p < 0.05$ according to the Student's *t*-test.

Supplementary Table S1. CONSORT 2010 checklist of information in this report

Section/Topic	Item No.	Checklist item	Reported on page No.
Title and abstract	1a	Identification as a randomized trial in the title	Title
	1b	Structured summary of trial design, methods, results, and conclusions (for specific guidance see CONSORT for abstracts)	Abstract
Introduction			
Background and objectives	2a	Scientific background and explanation of rationale	1. Introduction
	2b	Specific objectives or hypotheses	1. Introduction
Methods			
Trial design	3a	Description of trial design (such as parallel, factorial) including allocation ratio.	2.2.3. Study design
	3b	Important changes to methods after trial commencement (such as eligibility criteria), with reasons	Not applicable
Participants	4a	Eligibility criteria for participants	2.2.1. Participants
	4b	Settings and locations where the data were collected	2.2.1. Participants
Interventions	5	The interventions for each group with sufficient details to allow replication, including how and when they were actually administered	2.2.2. Test samples
Outcomes	6a	Completely defined pre-specified primary and secondary outcome measures, including how and when they were assessed	2.2.4. Outcome
	6b	Any changes to trial outcomes after the trial commenced, with reasons	Not applicable
Sample size	7a	How sample size was determined	2.2.10. Sample size

	7b	When applicable, explanation of any interim analyses and stopping guidelines	Not applicable
Randomization: Sequence generation	8a	Method used to generate the random allocation sequence	2.2.11. Randomization
	8 b	Type of randomization; details of any restriction (such as blocking and block size)	2.2.11. Randomization
Allocation concealment mechanism	9	Mechanism used to implement the random allocation sequence (such as sequentially numbered containers), describing any steps taken to conceal the sequence until interventions were assigned	2.2.11. Randomization
Implementation	10	Who generated the random allocation sequence, who enrolled participants, and who assigned participants to interventions	2.2.11. Randomization
Blinding	11a	If done, who was blinded after assignment to interventions (for example, participants, care providers, those assessing outcomes) and how	2.2.11. Randomization
	11b	If relevant, description of the similarity of interventions	Not applicable
Statistical methods	12a	Statistical methods used to compare groups for primary and secondary outcomes	2.2.12. Statistical Analysis
	12b	Methods for additional analyses, such as subgroup analyses and adjusted analyses	3.2.8. Stratified analysis, 4. Discussion
Results			
Participant flow (a diagram is strongly recommended)	13a	For each group, the numbers of participants who were randomly assigned, received intended treatment, and were analyzed for the primary outcome	3.2.1. Participants, Figure 2

	13b	For each group, losses and exclusions after randomization, together with reasons	3.2.1. Participants, Figure 2
Recruitment	14a	Dates defining the periods of recruitment and follow-up	2.2.3. Study design, 3.2.1. Participants
	14b	Why the trial ended or was stopped	Not applicable
Baseline data	15	A table showing baseline demographic and clinical characteristics for each group	3.2.2. Background of participants, Table 2
Numbers analyzed	16	For each group, number of participants (denominator) included in each analysis and whether the analysis was by original assigned groups	3.2.1. Participants, Figure 2
Outcomes and estimation	17a	For each primary and secondary outcome, results for each group, and the estimated effect size and its precision (such as 95% confidence interval)	3. Results
	17b	For binary outcomes, presentation of both absolute and relative effect sizes is recommended	3. Results
Ancillary analyses	18	Results of any other analyses performed, including subgroup analyses and adjusted analyses, distinguishing pre-specified from exploratory	3.2.8. Stratified analysis, 4. Discussion
Harms	19	All important harms or unintended effects in each group (for specific guidance see CONSORT for harms)	3.2.9. Safety Assessment
Discussion			
Limitations	20	Trial limitations, addressing sources of potential bias, imprecision, and, if	4. Discussion

		relevant, multiplicity of analyses	
Generalisability	21	Generalisability (external validity, applicability) of the trial findings	4. Discussion
Interpretation	22	Interpretation consistent with results, balancing benefits and harms, and considering other relevant evidence	4. Discussion
Other information			
Registration	23	Registration number and name of trial registry	2.2.3. Study Design
Protocol	24	Where the full trial protocol can be accessed, if available	2.2.3. Study Design
Funding	25	Sources of funding and other support (such as supply of drugs), role of funders	Funding

Supplementary Table S2. Exclusion criteria

No.	Exclusion criteria
1	Participants suffering from, undergoing treatment for, or with a history of serious diseases, such as diabetes, kidney/liver disease, heart disease or thyroid disease, adrenal disease, and other metabolic diseases.
2	Participants with chronic diseases and who take medication on a daily basis.
3	Participants who have been diagnosed with dry mouth.
4	Participants who are unable to abstain from taking supplement, food for specified health use or functional food, or health food that may affect immune function.
5	Participants who are unable to abstain from taking food that containing lactic acid bacteria, Bifidobacterium, oligosaccharides, or viable bacteria during the study period.
6	Participants who consistently drink more than the appropriate amount of alcohol.
7	Participants who are unable to abstain from alcohol for 2 days prior to the screening test and each test.
8	Participants with food allergies.
9	Participants who take or plan to take medicine for seasonal allergic rhinitis (pollen allergy).
10	Participants with digestive diseases affecting digestion and absorption and those with a history of digestive surgery (excluding appendicitis).
11	Participants who tend to get diarrhea by taking dairy products.
12	Participants who are pregnant women, women who intend to become pregnant during the research period, and women who are breastfeeding.
13	Participants who are judged to be inappropriate as research participants based on blood tests results obtained during the screening tests.
14	Participants who have a history or current condition of drug or alcohol dependence.
15	Participants who are participating in research involving the ingestion of other foods or the use of other medicines or those who have participated in or are willing to participate in other clinical research within 1 month of obtaining consent.
16	Participants who are judged to be inappropriate as research participants by the principal investigator.
17	Participants who smoke 21 or more cigarettes a day.
18	Participants who plan to receive the influenza vaccine from 3 weeks before ingestion to the end of the ingestion period.
19	Participants who plan to receive the coronavirus disease 2019 (COVID-19) vaccine

	during the ingestion period.
20	Participants who work on night shift.
21	Participants who plan to travel abroad, including overseas travel, during the study.
22	Participants who have donated more than 200 ml of blood within 1 month or 400 ml of blood within 3 months prior to the date of obtaining consent, or those who have donated blood components.

Supplementary Table S3. Instructions followed by the participants during the study

No.	Observance
1	Intake the test samples as instructed.
2	Do not allow other persons to intake the test samples.
3	Avoid drinking alcohol 2 days before the test.
4	Avoid taking any food and drinks after 21:00 on the day before the test (only water was permitted).
5	Avoid taking water 1 h before the test and until the end of the test.
6	Avoid smoking until the end of the test on the day of the test.
7	Avoid any dental treatment 2 days before the test.
8	Maintain your regular lifestyle, such as food and exercise (to avoid undereating, overeating, overexercising, and traveling abroad).
9	Avoid taking more food or drinks, including caffeine, than usual.
10	Avoid the use and/or the intake of medicines, supplements, and/or healthy food (including Food for Specified Health Uses and Foods with Functional Claims) that may influence the immune system.
11	Avoid the intake of food containing viable bacteria, such as lactic acid bacteria, Bifidobacteria, and natto (fermented soybeans) bacteria, and/or enhanced with oligosaccharides.
12	Avoid donation of blood and/or blood components.
13	Avoid the overconsumption of alcohol (up to 20 g alcohol/day).
14	Use medicines after getting the permission of the principal investigator (except in case of emergency).
15	Keep a daily record of the test sample consumption, defecation, ingestion of healthy food, usage of medicine, and physical health questionnaire.

Supplementary Table S4. Comparison of pDC activity (CD86 and HLA-DR expression)

Parameter	Week	Group	*n*	Measured value			
				**Mean**	**±**	**SD**	***p*-Value**
CD86	0	LG2055	95	1158.0	±	235.6	0.814
		Placebo	96	1172.6	±	311.8	
	6	LG2055	95	1033.4	±	212.8	0.582
		Placebo	95	1025.5	±	236.8	
	12	LG2055	95	870.7	±	190.1	0.359
		Placebo	96	844.0	±	164.9	
HLA-DR	0	LG2055	95	19429.7	±	4956.5	0.547
		Placebo	96	18998.7	±	4916.8	
	6	LG2055	95	19368.2	±	4603.2	0.305
		Placebo	95	18704.7	±	4274.0	
	12	LG2055	95	22231.3	±	5094.1	0.428
		Placebo	96	21666.9	±	4716.7	

Supplementary Table S5. Comparison of immunological markers

Parameter	Week	Group	*n*	Measured value			
				Mean	±	SD	*p*-Value
Salivary sIgA (μg/min)	0	LG2055	95	108.6	±	51.5	0.565
		Placebo	96	111.9	±	52.7	
	6	LG2055	95	115.9	±	53.8	0.972
		Placebo	95	120.3	±	68.2	
	12	LG2055	95	114.4	±	56.9	0.888
		Placebo	96	118.5	±	79.0	
Serum IgA (mg/dL)	0	LG2055	95	211.1	±	79.9	0.154
		Placebo	96	263.4	±	363.7	
	6	LG2055	95	212.9	±	82.8	0.175
		Placebo	95	266.7	±	387.8	
	12	LG2055	95	206.6	±	80.5	0.124
		Placebo	96	259.6	±	362.5	
Serum IgG (mg/dL)	0	LG2055	95	1188.5	±	218.6	0.690
		Placebo	96	1175.9	±	218.9	
	6	LG2055	95	1180.9	±	223.6	0.784
		Placebo	95	1172.0	±	218.9	
	12	LG2055	95	1194.3	±	223.4	0.896
		Placebo	96	1190.1	±	224.0	
NK cell activity (%)	0	LG2055	38	59.1	±	16.9	0.371
		Placebo	39	62.9	±	19.4	
	6	LG2055	38	66.6	±	14.6	0.349
		Placebo	39	70.1	±	17.8	
	12	LG2055	38	67.9	±	14.5	0.714
		Placebo	39	69.2	±	17.7	

Supplementary Table S6. Effects of LG2055 intake on fecal microbiota

Genus	Group	*n*	0 week			12 weeks		
			**Mean**	**±**	**SD**	**Mean**	**±**	**SD**
Lactobacillus (%)	LG2055	95	0.02	±	0.09	0.03	±	0.10 **, #
	Placebo	96	0.03	±	0.22	0.02	±	0.15
Butyricimonas (%)	LG2055	95	0.07	±	0.13	0.11	±	0.20 ##
	Placebo	96	0.11	±	0.26	0.11	±	0.21
Agathobacter (%)	LG2055	95	1.16	±	1.61	1.46	±	1.90 #
	Placebo	96	1.08	±	1.60	1.38	±	2.19

*Significant difference between two groups (** $p < 0.01$).

#Significant difference within the group (# $p < 0.05$, ## $p < 0.01$).

Supplenentary Table S7. Comparison of pDC activity (CD86 and HLA-DR expression) (stratified analysis)

Parameter	Week	Group	*n*	Measured value			
				**Mean**	**±**	**SD**	***p*-Value**
CD86	0	LG2055	57	1169.3	±	262.4	0.623
		Placebo	55	1178.8	±	342.7	
	6	LG2055	57	1033.3	±	212.8	0.543
		Placebo	55	1011.0	±	214.7	
	12	LG2055	57	887.7	±	203.6	0.025*
		Placebo	55	806.1	±	146.7	
HLA-DR	0	LG2055	57	19248.1	±	5261.2	0.202
		Placebo	55	18026.0	±	4789.9	
	6	LG2055	57	19213.3	±	4463.5	0.110
		Placebo	55	17914.1	±	4057.7	
	12	LG2055	57	22496.9	±	4930.2	0.090
		Placebo	55	20946.1	±	4640.7	

*Significant difference between the two groups ($p < 0.05$).

Supplementary Table S8. Bacterial composition of each cluster (top 10 genera)

Genus	Cluster 1	Cluster 2	Cluster 3
Bacteroides	15.5%	43.4%	28.7%
Prevotella	27.4%	0.5%	1.9%
Faecalibacterium	6.1%	5.8%	4.8%
Parabacteroides	4.0%	4.4%	4.1%
Bifidobacterium	1.6%	4.2%	4.7%
family *Lachnospiraceae* (unclassified genus)	2.0%	3.5%	2.3%
Phascolarctobacterium	3.3%	2.2%	1.7%
Lachnoclostridium	2.2%	2.5%	1.5%
Sutterella	2.2%	2.1%	1.8%
Fusobacterium	1.2%	1.9%	1.1%

Supplementary Table S9. Comparison of pDC activity (CD86 expression) in each cluster

<table>
<tr><th rowspan="2">Cluster</th><th rowspan="2">Week</th><th rowspan="2">Group</th><th rowspan="2">n</th><th colspan="4">Measured value</th></tr>
<tr><th>Mean</th><th>±</th><th>SD</th><th>p-Value</th></tr>
<tr><td rowspan="6">1</td><td rowspan="2">0</td><td>LG2055</td><td>26</td><td>1109.6</td><td>±</td><td>190.3</td><td rowspan="2">0.642</td></tr>
<tr><td>Placebo</td><td>11</td><td>1293.1</td><td>±</td><td>570.3</td></tr>
<tr><td rowspan="2">6</td><td>LG2055</td><td>26</td><td>962.7</td><td>±</td><td>137.6</td><td rowspan="2">0.173</td></tr>
<tr><td>Placebo</td><td>11</td><td>1099.4</td><td>±</td><td>282.2</td></tr>
<tr><td rowspan="2">12</td><td>LG2055</td><td>26</td><td>882.4</td><td>±</td><td>230.5</td><td rowspan="2">0.046 *</td></tr>
<tr><td>Placebo</td><td>11</td><td>744.3</td><td>±</td><td>141.8</td></tr>
<tr><td rowspan="6">2</td><td rowspan="2">0</td><td>LG2055</td><td>45</td><td>1155.4</td><td>±</td><td>221.0</td><td rowspan="2">0.649</td></tr>
<tr><td>Placebo</td><td>43</td><td>1191.3</td><td>±</td><td>285.2</td></tr>
<tr><td rowspan="2">6</td><td>LG2055</td><td>45</td><td>1051.7</td><td>±</td><td>248.7</td><td rowspan="2">0.607</td></tr>
<tr><td>Placebo</td><td>43</td><td>1044.1</td><td>±</td><td>277.4</td></tr>
<tr><td rowspan="2">12</td><td>LG2055</td><td>45</td><td>846.7</td><td>±</td><td>171.0</td><td rowspan="2">0.238</td></tr>
<tr><td>Placebo</td><td>43</td><td>890.6</td><td>±</td><td>166.8</td></tr>
<tr><td rowspan="6">3</td><td rowspan="2">0</td><td>LG2055</td><td>24</td><td>1215.3</td><td>±</td><td>296.5</td><td rowspan="2">0.170</td></tr>
<tr><td>Placebo</td><td>42</td><td>1121.8</td><td>±</td><td>236.1</td></tr>
<tr><td rowspan="2">6</td><td>LG2055</td><td>24</td><td>1075.8</td><td>±</td><td>195.0</td><td rowspan="2">0.117</td></tr>
<tr><td>Placebo</td><td>42</td><td>987.6</td><td>±</td><td>169.8</td></tr>
<tr><td rowspan="2">12</td><td>LG2055</td><td>24</td><td>902.9</td><td>±</td><td>177.8</td><td rowspan="2">0.059</td></tr>
<tr><td>Placebo</td><td>42</td><td>822.4</td><td>±</td><td>155.5</td></tr>
</table>

*Significant difference between two groups ($p < 0.05$).
